# Monthly Report of Diseases

**Published:** 1800-05

**Authors:** 


### MONTHLY REPORT of DISEASES,

### Admitted under the Care of the PHYSICIANS of the FINSBURY DISPENSARY, St. John's Square, Clerkenwell.

*The District, in which the Patients of the Finsbury Dispensary are visited, comprehends the Parishes of St. James, and of St. John, Clerkenwell; of St. Luke; of St. Sepulchre within and without; of St. Bartholomew, the Great and the Less; the Liberties of the Rolls, and of Glass-House Yard; the Town of Islington; the Parishes of St. Pancras; of St. Andrew, Holborn; and of St. George the Martyr, Queen's-square. This Tract of Ground may properly enough be termed, a North Western District of the Metropolis.*

LIST of DISEASES, &c. from March 20, to April 20.

| | No. of Cases. | | No. of Cases. |
|---|---|---|---|
| Continued Fever - - | 16 | Cephalæa - - - - - | 5 |
| Scarlet Fever - - - - - | 2 | Nephralgia Calculosa - - | 1 |
| Measles - - - - - | 1 | Pleurodyne - - - - - | 3 |
| Sore Throat - - - | 4 | Hydrops - - - - - - | 4 |
| Hæmoptysis - - - - - | 4 | Hæmorrhois - - - - - | 3 |
| Pulmonary Complaints without Fever - - - - - | 53 | Hysteria - - - - - - | 3 |
| Phthisis Pulmonalis - - | 12 | Paralysis - - - - - | 3 |
| Dysentery - - - - - - | 3 | Apoplexy - - - - - - | 1 |
| Diarrhœa - - - - - - | 4 | Schrophula - - - - - | 2 |
| Chlorosis and Amenorrhœa | 29 | Colica Pictonum - - - | 1 |
| Leucorrhœa - - - - - - | 7 | Hypochondriasis - - - | 1 |
| Menorrhagia - - - - - | 6 | Insanity - - - - - - | 2 |
| Asthenia - - - - - - | 10 | Hooping Cough - - - | 4 |
| Dyspepsia - - - - - - | 6 | Rheumatism - - - - - | 4 |
| Enterodynia - - - - - - | 2 | Febricula - - - - - - | 4 |
| Peritonitis - - - - - - | 1 | Febris Mesenterica - - | 3 |
| Constipatio - - - - - | 1 | Vermes - - - - - - | 8 |
| Vertigo - - - - - - | 2 | Fever Infantilis - - - - | 6 |
| | | Chronic Cutaneous Diseases | 15 |

In the above catalogue of diseases, the cases of amenorrhœa bear more than their usual proportion. Of these, some evidently arose from a mental affection; others, from excessive corporeal fatigue; but the greater number from the partial application plication of cold, eſpecially to the feet. In ſeveral inſtances, riding on the box of a hackney-coach was preſcribed; and in the only one in which the advice was faithfully obſerved, it was attended with evident and ſpeedy advantage. The motion of ſuch a vehicle, over the rough pavement of London, affords no inconſiderable exerciſe, without demanding any degree of that voluntary exertion, of which, in many caſes of this diſeaſe, the patient ſeems altogether incapable.

Thoſe pains in the lower part of the back, with which female patients between forty and fifty years of age are ſo frequently afflicted, have, nearly without exception, been relieved by the emplaſtrum thuris compoſitum. Whether this operates *altogether* immediately on the body, or, *in part*, through the imagination, it may not be eaſy to aſcertain; but a vaſt number of experiments authorize us in conſidering it as, in ſome way or other, almoſt a *ſpecific* in the diſeaſe.

A caſe of hooping-cough occurred in a man of ſeventy-one years of age. He had never any cough before. He ſaid that his child had been afflicted with the ſame complaint, and that he had received from him the infection. The peculiar ſymptoms of the diſeaſe were diſtinctly and ſtrongly marked. So favourable a hope, with regard to the event of the diſorder, could not be entertained in this inſtance, as in thoſe where it occurs at the uſual period, eſpecially as the patient was, independently of his age, more than commonly enfeebled and emaciated. In ſpite, however, of theſe circumſtances, after the application of a bliſter to his breaſt, mucilaginous mixtures, medicines gently opening, and opiates at night, the cough in a ſhort time completely diſappeared, leaving only a degree of weakneſs, which might be expected from an advanced period of life, and the operation of ſo violent a diſorder.

In one of the patients afflicted with inſanity, it evidently aroſe from the intemperate and long continued uſe of inebriating liquors. Moſt of the diſeaſes, indeed, that prevail amongſt the poor in London, originate from an exceſs of ſtimulus, connected with a defect of nouriſhment. This circumſtance conſtitutes one of the principal obſtacles to the ſucceſs of diſpenſary practice. To a patient, whoſe only change of diet is from tea to ſpirits, what real advantage is to be expected from any pharmaceutical preparations? The draughts of the apothecary's ſhop, when oppoſed to thoſe of the gin ſhop, cannot have any effectual or ſalutary operation.

In addition to this circumſtance, there is another difficulty with which a practitioner amongſt the poor is obliged to contend, ariſing from the little confidence that is to be placed in the attention of the perſons about the patient, and in the faithful ful adminiftration of the remedies which are prefcribed. This remark applies even to thofe that are bound together by the neareft ties.

In a cafe of peritoneal inflammation, leeches were ordered, and a trifling fum was given to the wife of the patient for the purchafe of them, which, however, inftead of procuring with it a remedy, that fhe was informed was neceffary to the life of her hufband, fhe expended in inebriating draughts.

Accordingly, when the phyfician called the fucceeding morning, he found the man and woman both lying on the fame bed; the one dead, and the other in the laft ftage of intoxication.

There is no perfon, perhaps, who is apt to form fo low an eftimate of the value of human exiftence, as a medical man practifing amongft the poor, efpecially amongft the poor of a great city. But it is not impoffible that he may exaggerate the excefs of their fufferings, by combining, as it is natural for him to do, their external ftate, with thofe feelings which he has acquired from very different circumftances and education. As the horrors of the grave affect only the living, fo the miferies of poverty exift principally, perhaps, in the imagination of the affluent.

The labour of the poor man relieves him at leaft from the burden of fafhionable ennui; and the conftant preffure of phyfical inconveniences, from the more elegant, but furely not lefs intolerable diftreffes of a refined and romantic fenfibility.

Even thofe fuperior intellectual advantages of education, to which the more opulent are almoft exclufively admitted, may, in fome cafes, open only new avenues to forrow. The mind, in proportion as it is expanded, expofes a larger furface to impreffion.

